# Effect of Airborne Particle Abrasion on Microtensile Bond Strength of Total-Etch Adhesives to Human Dentin

**DOI:** 10.1155/2017/2432536

**Published:** 2017-12-17

**Authors:** Maurizio D'Amario, Chiara Piccioni, Stefano Di Carlo, Francesca De Angelis, Silvia Caruso, Mario Capogreco

**Affiliations:** ^1^Unit of Restorative Dentistry, Endodontics and Oral Pathology, Department of Life, Health and Environmental Sciences, Dental Clinic, University of L'Aquila, L'Aquila, Italy; ^2^Department of Oral and Maxillofacial Sciences, Sapienza University of Rome, Rome, Italy

## Abstract

Aim of this study was to investigate a specific airborne particle abrasion pretreatment on dentin and its effects on microtensile bond strengths of four commercial total-etch adhesives. Midcoronal occlusal dentin of extracted human molars was used. Teeth were randomly assigned to 4 groups according to the adhesive system used: OptiBond FL (FL), OptiBond Solo Plus (SO), Prime & Bond (PB), and Riva Bond LC (RB). Specimens from each group were further divided into two subgroups: control specimens were treated with adhesive procedures; abraded specimens were pretreated with airborne particle abrasion using 50 *μ*m Al_2_O_3_ before adhesion. After bonding procedures, composite crowns were incrementally built up. Specimens were sectioned perpendicular to adhesive interface to produce multiple beams, which were tested under tension until failure. Data were statistically analysed. Failure mode analysis was performed. Overall comparison showed significant increase in bond strength (*p* < 0.001) between abraded and no-abraded specimens, independently of brand. Intrabrand comparison showed statistical increase when abraded specimens were tested compared to no-abraded ones, with the exception of PB that did not show such difference. Distribution of failure mode was relatively uniform among all subgroups. Surface treatment by airborne particle abrasion with Al_2_O_3_ particles can increase the bond strength of total-etch adhesives.

## 1. Introduction

It is well known that dentin has several intrinsic features making in complex mode the adhesion of resinous materials (wet, inhomogeneity, and smear layer). It is already accepted that, for total-etch adhesives, the interaction is mainly micromechanical: it is essential to make hybrid layer and resin tags to obtain a reliable dentin adhesion. Hybrid layer is made by the resin interdiffusion into the collagen, previously exposed by acid etching [1]; to make resin tags into dentinal tubes, patency condition of dentinal tubules themselves is needed [2].

With the aim of improving the interaction between resin and dentin, several dentinal pretreatments techniques have been tentatively introduced [3, 4]. Besides to be the requirement for micromechanical adhesion, pretreatments can be used with the aim of removing debris that can impair the final bonding restoration [5]. In fact, they can impair etching quality or may even inhibit the resinous monomers polymerization [6].

Starting from this assumption, it can be imagined that dentin surface cleaning may be essential to obtain better bonding between the interfaces [7, 8]. Several cleaning methods, both mechanical and chemical, have been proposed [7, 8]. Related to chemical cleaning techniques, the most common technique includes the chlorhexidine digluconate, sodium hypochlorite, hydrogen peroxide, and ethylene diamine tetra acetic acid use (EDTA) [9]. Laser techniques were proposed as pretreatments [10, 11], but microtensile bond strength measurements reports after laser pretreatment are confusing and contradictory. Some studies reported higher bond strengths to laser-prepared dentin [12–14]. Conversely, others papers testified significantly lower bond strengths [15–17] or no significant differences [18].

Airborne particle abrasion (APA) with aluminum oxide is a mechanical pretreatment technique: it is a cost-effective method for surface roughening that can provide an extra ultrafine mechanical retention [19, 20]. Currently, airborne abrasion is most commonly used to generate roughness in ceramic or composite restorations and increase the bond surface area, which might improve the bonding values [21, 22]. The rationale behind this procedure indicates that this method can also improve dentin bonding [19, 20]. Therefore, the evaluation of the effects of this procedure on bond strengths of different bonding agents to human dentin is mandatory with the aim of establishing a possible protocol [20]. Some studies examined bond strengths using self-etch adhesives after APA pretreatment, but a comparative evaluation testing total-etch adhesives is still missing.

On these bases, the aim of this* in vitro* study was to investigate the effect of direct dentin airborne particle abrasion using aluminum oxide (50 *μ*m particles) on the microtensile bond strengths of four commercial total-etch adhesives. The null hypothesis tested was that the bond strengths values would not be affected by pretreatment procedure.

## 2. Materials and Methods

Forty freshly extracted third human molars, free of cracks, caries, and restorations upon visual inspection, were selected for the study. Any residual soft tissue and debris were removed from the roots with a scaler. The teeth were then rinsed with water and stored in an aqueous solution of 0.5% chloramine T at 4°C for not longer than three months until the start of the experiment.

Each crown was sectioned perpendicularly to its longitudinal axis, 4 mm from the cement-enamel junction, using a low-speed diamond saw (Micromet M, Remet; Bologna, Italy) under copious PBS spray, to expose a flat dentin surface in the middle crown portion; then teeth were stored. Each surface was then ground with 180-grit silicon carbide (SiC) paper on a polisher (Polimet, Buehler Ltd., Lake Bluff, IL, USA), under running water for 30 s to produce and standardize the smear layer thickness on the dentin surface. The bonding surfaces were then examined under a stereomicroscope (Nikon SMZ10, Tokyo, Japan) to ensure that they were free from residual enamel. If any enamel remained, the surface was ground again until all the enamel was removed. Teeth were then randomly divided into four groups (*n* = 10 for each group).

Four commercially available etch-and-rinse adhesive systems (Table 1) were used in the experiments in order to form four groups, as follows: FL, SO, PB, and RB, in which the adhesive systems applied on dentin surface were OptiBond FL (Kerr Corporation), OptiBond Solo Plus (Kerr Corporation), Prime & Bond (Densply Caulk), and Riva Bond LC (SDI Limited), respectively.

Each group was randomly divided into two subgroups: control (C) (treated with adhesive procedures) and abraded (A) (APA with aluminum oxide before to be treated with adhesive procedures), respectively (5 teeth/subgroup).

Abraded groups surfaces were sandblasted with 50 *μ*m Al_2_O_3_ (Korox; Bego, Bremen, Germany) for 10 seconds, 5 cm away from surface with angle of approximately 90°, using intraoral air abrasion device (Micerium, Avegno, Genoa, Italy) at a pressure of 2 bar (“soft-sandblasting procedure”) [23, 24]. They were then rinsed with water for 15 seconds and dried for 5 seconds.

All specimens were etched for 15 seconds with the phosphoric acid gel provided by the respective manufacturers (Table 1) and were subsequently washed using a water spray for at least 15 seconds. Excess water was blot dried from the dentin surface with a wet cotton pellet, leaving the surface visibly moist.

In each group, the adhesive system was applied according to the manufacturers' instructions (Table 1) and then light cured for 20 seconds with a curing light (Bluephase C8, with 800 mW/cm^2^ output, Ivoclar Vivadent AG, Schaan, Liechtenstein).

Each adhesive system (FL, SO, PB, and RB) was applied to respective group, directly into a disposable microbrush and gently rubbed on dentin surfaces, avoiding pooling (Table 1). Following adhesive application, four increments of resin composite (Herculite XRW Ultra-A2 Dentin; Kerr Corporation) of about 1 mm were built up and individually light activated for 40 seconds (Bluephase C8, Ivoclar Vivadent AG) on each specimen. After restorative procedures described above, specimens were stored in distilled water at 37°C for 24 h and then underwent 30,000 thermal cycles in deionized water from 5°C to 55°C, with a 30-second dwelling time and 5-second transfer between temperature baths (LTC100; LAM technologies Electronic Equipment, Firenze, Italy) [25, 26].

Specimens were then sectioned perpendicular to the adhesive interface with a diamond saw (Micromet M, Remet; Bologna, Italy) under PBS cooling/lubrication to produce beams with adhesive area of approximately 1 mm^2^.

Six beams from the central part of each specimen were obtained per tooth. A total of 30 beams (*n* = 30) were subsequently used for each subgroup. Microtensile bond strength test was performed for each beam.

### 2.1. Microtensile Bond Strength Test

Beams were attached to the flat grips of a microtensile testing device using a cyanoacrylate cement and stressed in a universal testing machine (LMT 150; LAM technologies Electronic Equipment), with a cross-head speed of 0.5 mm/min until failure. A chain of 2 links was interposed between the device and the upper clamp of the testing machine. Once tested, specimens were removed from the testing devices and the cross-sectional areas of the fracture sites were measured with a digital caliper (series 500 Caliper; Mitutoyo America, Aurora, IL, USA) to calculate the ultimate tensile bond strength expressed in MPa.

### 2.2. Mode of Failure

After the *μ*-TBS test, the dentin sides of fractured specimens were mounted on aluminum stubs, gold-sputter coated and observed by SEM (EVO 50 XVP LaB6, Carl Zeiss, Cambridge, UK) at 100x or higher magnification for fracture mode determination. Failure modes were classified into 4 different types: Type 1: adhesive fracture between adhesive agent and dentin; Type 2: adhesive fracture between adhesive agent and dentin plus partial cohesive fracture in the composite restoration or dentin (mixed failure); Type 3: cohesive fracture in dentin; Type 4: cohesive fracture in the composite restoration [27].

### 2.3. Statistical Analysis

Normal data distribution was tested by Shapiro-Wilk test. Microtensile bond strength (MPa) was compared using two-way ANOVA and multiple comparisons were tested post hoc by Tukey's HSD. Significance level was set at *p* < 0.05. Statistical analysis and graph plots were performed using the R packages “stats” and “ggplot” (v3.0.3, R Foundation for Statistical Computing, Vienna, Austria).

## 3. Results

Results of ANOVA analysis and post hoc Tukey's comparisons are presented in Table 2 (*n* = 30). Two-way ANOVA showed that the adhesive system used and the pretreatment protocol adopted significantly affected the bond strength (*p* < 0.001). A significant interaction was recorded between the two factors (*p* < 0.05). The null hypothesis tested was thus rejected. Briefly, overall comparison showed significant increase in bond strength (*p* < 0.001) between abraded (32.51 ± 8.78 MPa) and not abraded specimens (19.24 ± 7.47 MPa), independently of brand. Intrabrand comparison showed statistical increase in terms of required MPa when abraded specimens were tested compared to not abraded ones, with the exception of PB that did not show such difference. Not abraded RB specimens showed significantly lower compared to the other not abraded tested products. There were found no differences in terms of bond strength among abraded specimens. No pretest failures were recorded.

**Figure 1:**
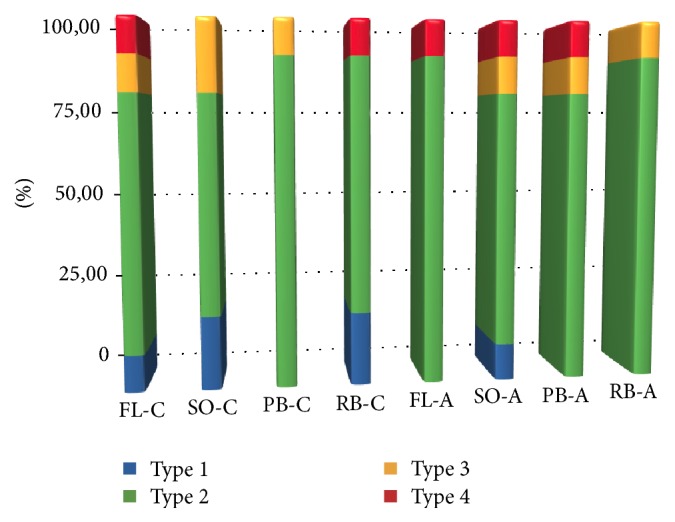
Distribution (%) of failure mode in experimental groups after microtensile bond strength test.* Type 1*: adhesive fracture between adhesive agent and dentin;* Type 2*: adhesive fracture between adhesive agent and dentin plus partial cohesive fracture in the composite restoration or dentin (mixed failure);* Type 3*: cohesive fracture in dentin;* Type 4*: cohesive fracture in the composite restoration.

**Figure 2:**
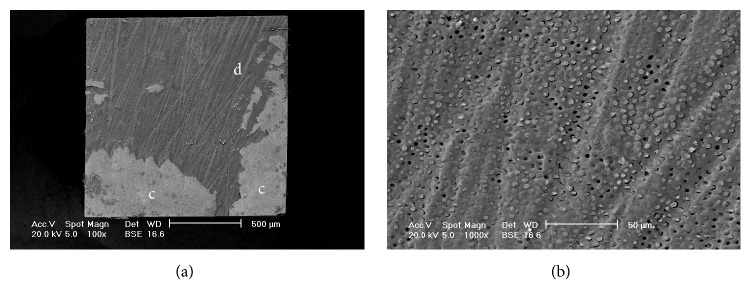
Scanning electron microscope (SEM) at backscattered electrons images of a Type 2 failure (adhesive fracture between adhesive agent and dentin plus partial cohesive fracture in the composite restoration) (PB-A). (a) Magnification ×100. d: dentin and c: composite. (b) Dentinal tubules are evident at higher magnification (×1000).

**Figure 3:**
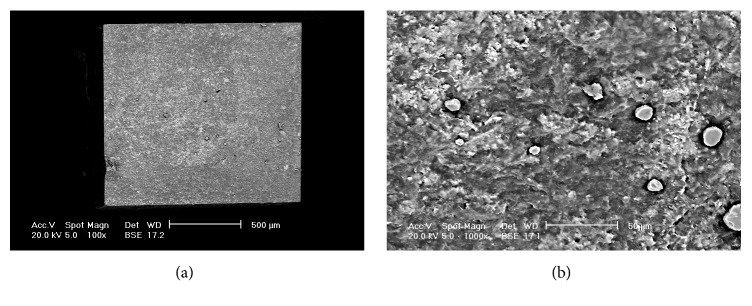
Scanning electron microscope (SEM) at backscattered electrons images of a Type 4 failure (cohesive fracture in the composite restoration) (FL-A). (a) Magnification ×100. (b) Resin composite structure at higher magnification (×1000).

Figure 1 displays the results of the failure mode analysis. Type 2 failure (adhesive fracture between dentin and adhesive agent plus partial cohesive fracture in dentin or composite restoration) was the most prevalent failure mode in all subgroups (Figure 2). The other types of failure mode were relatively uniform among all subgroups (Figure 3).

## 4. Discussion

The ability to remove the smear layer on dentin is a well-known effect of phosphoric acid application. In contrast, there are only a few documented effect of APA on dentin surfaces [19, 28]. A recent paper documented as APA treatment can produce a rough surface on dentin, preserving the original diameter of dentin tubule orifices and, consequently, the amount of available intertubular dentin [20]. The results of the present study corroborate with the finding of these previous reports [19, 20, 28], showing a significant increase of bond strength in abraded specimens. Only in PB groups, the air APA did not affect the adhesive performance.

Artificial aging, performed using critical thermal cycles, could have influenced the mean values of microtensile bond strength registered, as confirmed by literature [25, 26]. Although thermal cycling is one of the most widely aging method used, there is an apparent lack of a standardized protocol [25]. The choice of parameters for thermal cycling (temperature, dwell time, and number of cycles) seems to be commonly chosen on the basis of convenience [25]. In the present study, 30,000 cycles of thermocycling was chosen based on the results of a recent study that showed that thermal cycling can affect, with a progressive nonlinear decrease, the mechanical properties of resin composites [26]. In particular, Morresi et al. [26] demonstrated that a short thermal cycling protocol (15,000 cycles) was not able to affect most of the tested specimens, which have been influenced by a greater number of cycles (30,000 or more).

According to the current literature, the increased adhesive strength registered in abraded specimens could have been obtained with the increase on micromechanical retention and wettability of the adhesive systems [29]. The following water rinsing and acid etching could remove Al_2_O_3_ particles, leaving a positive effect on penetration of adhesive to dentin which could explain the higher bond strength of abraded group compared to the control group. The strength increase and dentin adhesion quality is shown by SEM images obtained after failure tests too: the predominant failure mode is not on the dentin-adhesive interface, but it is mixed with fracture on the adhesive-restorative materials interface, and that could reflect the effectiveness of this bond.

The use of aluminum oxide was chosen for several reasons: aluminum is one of the lowest valence metals, not commonly found in humans. Its oxide is highly insoluble and, unlike many other aluminum salts, nontoxic; that results in excellent biocompatibility [30]. It was showed that aluminum oxide sandblasted enamel provides a reliable method for increasing the microtensile bond strength of composite resins to enamel [31]. Several authors have used different kind of APA to evaluate effects on the bond strength. Carvalho et al. [32] used experimental niobo-phosphate bioactive glass and concluded that it did not interfere with the immediate bonding performance of self-etching and self-adhesive cements. The authors proposed that air abrasion with experimental bioactive glass is not a way to enforce bond strength but that this pretreatment powder did not interfere with the performance of the restorative materials and is a promising technique to participate in the formation of a “hybrid bioactive layer” [32].

A recent study [33] showed that there are no significant differences between different dentin pretreatments (air abrasion and sonic technique) in microtensile bond strength. The study concluded that the surface roughness is not the only factor influencing the bonding but it is important to consider also the chemical composition of dentin surface and chemical parameters [34]. In the study, in fact, the authors used self-etch adhesive that incorporates the smear layer into the hybrid layer and formation of the resin tags into dentinal tubules that does not influence the bonding strength; in this way, the self-etch adhesive used could explain why air abrasion did not improve the bonding to dentin as a result in this study.

In a similar way, Yazici et al. [35] studied the different pretreatment methods effect on dentin bond strength of a one-step self-etch adhesive. They stated that bond strength decrease with laser and acid pretreatments; instead the air abrasion did not affect the adhesive performance.

Mujdeci and Gokay [19] tested dentin and enamel bond strength after APA with several restorative materials; they concluded that the bond strength of all restorative materials to enamel and dentin showed increased with APA compared to the control groups. Authors explained that several reasons could be offered for these findings: the increased surface area, the type of smear layer, and the increase of the wettability of tooth structure.

According to Rafael et al. [20] the quality of the available intertubular dentin might be the key to achieve a reliable adhesion. They showed through SEM images the effect of air abrasion on dentin surface: dentin tubules orifices exposure and increase of roughness on the intertubular dentin that can enlarge the contact area for adhesion.

Several authors propose the APA as a cleaning technique of dentin surface after the removal of an interim prosthesis too. Erkut et al. [7] tested some different cleansing treatment effects (microairborne-particle abrasion, alcohol, rubber-rotary instruments, and desiccating agent) on the bond strength of composite resin restoration: they found that the highest bond strength is achieved with the microairborne-particle abrasion technique. They conclude that the results could be attributed to the different surface texture obtained by different techniques.

In the present study, an evaluation of possible deleterious effects of abrasion on dentin surface was not investigated. Rafael et al. [20] reported some crack-like alterations on the tubule borders and on intertubular dentin and also a certain amount of debris on dentin surfaces (50 *μ*m Al_2_O_3_ particles, 60 psi, 5 seconds, and 5 mm). However, the APA protocol adopted in the present study (50 *μ*m Al_2_O_3_ particles, 2.0 bars, 10 seconds, and 5 cm) is considered quite mild (the tip of the intraoral air abrasion device is held 10 times further respect to the protocol by Rafael et al. [20]) and has already been tested on several dental materials [21, 23, 36].

Another possible limit of the present study is that the bond strength was evaluated without considering different pulpal pressure, though, as demonstrated in a precedent study, the presence of simulated pulpal pressure does not produce differences. Flury et al. [37] attained that there are no significant differences in bond strength between different kinds of pretreatment and between presence or absence of simulated pulpal pressure and any significant interaction of two factors.

## 5. Conclusions

In conclusion, surface treatment by APA with Al_2_O_3_ particles can increase the bond strength of total-etch adhesives to dentin.

## Figures and Tables

**Table 1:** Manufacturer, composition, and application mode of the adhesive systems tested.

Adhesive	Steps	Composition	Phosphoric acid gel	Application mode	Producer
*OptiBond FL* (FL)	3	Glass, oxide, chemicals, 2-hydroxyethyl methacrylate, ytterbium trifluoride, 3-trimethoxysilylpropyl methacrylate, 2-hydroxy-1,3-propanediyl bismethacrylate, alkali fluorosilicates (Na)	*Gel etchant* (37.5% phosphoric acid gel)	*OptiBond FL Prime* was applied with light brushing motion for 15 seconds and then air dried for 5 seconds; using the same applicator; *OptiBond FL Adhesive* was applied with light brushing motion for 15 seconds and air thinned for 3 seconds	Kerr Corporation, Orange, CA, USA

*OptiBond Solo Plus* (SO)	2	Ethyl alcohol, alkyl dimethacrylate resins, barium aluminoborosilicate glass, fumed silica (silicon dioxide), sodium hexafluorosilicate	*Gel etchant* (37.5% phosphoric acid gel)	*OptiBond Solo Plus* was applied with applicator tip for 15 seconds, using light brushing motion, and air thinned for 3 seconds	Kerr Corporation, Orange, CA, USA

*Prime & Bond NT* (PB)	2	Di- and trimethacrylate resins, PENTA (dipentaerythritol penta-acrylate monophosphate), nanofillers-amorphous silicon dioxide, photoinitiators, stabilizers, cetylamine hydrofluoride acetone	*Caulk 34% tooth conditioner gel *(34% phosphoric acid gel)	Using applicator tip, generous amounts of *Prime & Bond NT* adhesive to thoroughly wet all the tooth surfaces. The surfaces remained fully wet for 20 seconds; otherwise additional applications of adhesive were applied. Solvent was after that evaporated by methodically blowing with air syringe for at least 5 seconds in order to form adhesive surface with uniform glossy appearance	Dentsply Caulk, Milford, DE, USA

*Riva Bond LC* (RB)	2	Compartment 1: acrylic acid homopolymer, tartaric acid, 2-hydroxyethyl methacrylate, dimethacrylate cross-linker, acidic monomer Compartment 2: glass powder	*Super etch 37% *phosphoric acid etchant	Capsule was tapped twice on the bench and immediately mixed in an amalgamator for 10 seconds. Using a disposable applicator, *Riva Bond LC* was applied thinly over the surface of the cavity	SDI Limited, Bayswater, Australia

**Table 2:** Mean values (MPa (SD)) of microtensile bond strength for each analyzed subgroup (*n* = 30).

	C (control)	A (abraded)	*p*
FL	18.31 (6.72)^A^	35.51 (8.41)^A^	<0.001
SO	16.49 (4.61)^A^	32.60 (7.31)^A^	<0.001
PB	27.68 (4.98)^A^	33.36 (9.98)^A^	0.066
RB	14.47 (5.75)^B^	28.73 (7.06)^A^	<0.001

2-way ANOVA with Tukey's comparison. Letters for vertical comparisons.
